# Sodium-Glucose Cotransporter-2 Inhibitors in Anthracycline-Treated Cancer Patients: A Narrative Review of Emerging Cardioprotective Evidence

**DOI:** 10.7759/cureus.107433

**Published:** 2026-04-21

**Authors:** Elias Nabhan, Alexandrina-Paula Vana, Rida Khouri, Edouard Naoum Nehme, Nabil Poulos

**Affiliations:** 1 Cardiology, Centre Hospitalier Josephine Baker, Gonesse, FRA

**Keywords:** anthracyclines, cancer therapy-related cardiac dysfunction, cardio-oncology, cardiotoxicity, heart failure, sodium glucose co-transporter 2 inhibitors (sglt2i)

## Abstract

Randomized controlled trials (RCTs) evaluating the cardioprotective effects of sodium-glucose cotransporter-2 inhibitors (SGLT2i) on cancer therapy-related cardiac dysfunction (CTRCD) are scarce. This narrative review synthesizes the available evidence from six primary clinical studies (n=21,523), all of which are observational or non-randomized, and places these findings within their mechanistic and clinical context. Across studies, SGLT2i use is consistently associated with lower rates of cardiovascular events, including heart failure (HF) hospitalization and imaging-defined cardiac dysfunction. However, all estimates are subject to substantial residual confounding, including healthy user bias and immortal time bias, and should be considered hypothesis-generating rather than causal. The magnitude of observed associations exceeds what is mechanistically plausible based on established HF trials, further supporting the likelihood of bias. Current evidence therefore does not support routine clinical use of SGLT2i for CTRCD prevention. Three ongoing randomized trials will determine whether these observational signals translate into clinical benefit and define the role of SGLT2i in cardio-oncology practice.

## Introduction and background

Cardiovascular disease has emerged as the main non-cancer cause of long-term mortality in cancer survivors. As oncological outcomes continue to improve, this burden is expected to continue to grow [1,2]. Anthracyclines like doxorubicin, epirubicin, daunorubicin, and idarubicin are essential in the management of breast cancer, lymphoma, leukemia, and sarcoma. Clinically significant cancer therapy-related cardiac dysfunction (CTRCD) occurs in 5-18% of exposed patients, especially with symptomatic heart failure (HF), which may prompt premature discontinuation of life-prolonging cancer therapy [3,4].

The pathophysiology of anthracycline-induced cardiotoxicity (AIC) involves topoisomerase IIβ inhibition in cardiomyocytes, oxidative stress driven by anthracycline-iron complexes and reactive oxygen species, mitochondrial dysfunction, and progressive cardiomyocyte apoptosis [5,6]. These mechanisms are manifested as impaired contractile function, initially detectable as a reduction in global longitudinal strain (GLS) before an overt decline in left ventricular ejection fraction (LVEF) occurs.

Currently, cardioprotective strategies remain limited. Dexrazoxane reduces AIC through iron chelation but carries regulatory restrictions [7]. Beta-blockers and renin-angiotensin-aldosterone system (RAAS) inhibitors, evaluated in the PRADA [8], OVERCOME [9], MANTICORE [10], and CECCY [11] randomized trials, demonstrated modest LVEF preservation without definite CTRCD prevention.

Sodium-glucose cotransporter-2 inhibitors (SGLT2i) represent the most significant advance in HF pharmacology in a generation, and they are increasingly being investigated in non-traditional settings. The DAPA-HF [12], EMPEROR-Reduced [13], EMPEROR-Preserved [14], and DELIVER [15] trials established cardiovascular benefits across the spectrum of HF ejection fraction phenotypes, independent of diabetes status. Their cardioprotective mechanisms are pleiotropic: natriuresis and osmotic diuresis reducing cardiac preload and afterload; inhibition of the cardiac sodium-hydrogen exchanger (NHE1) reducing intracellular sodium and calcium overload; adenosine monophosphate-activated protein kinase (AMPK) activation promoting mitochondrial biogenesis and autophagic clearance of damaged organelles; NLR family pyrin domain-containing protein 3 (NLRP3) inflammasome suppression; and metabolic substrate optimization favoring ketone body utilization [16,17].

What makes SGLT2i interesting in the anthracycline context is the mechanistic overlap between these protective pathways and the known drivers of AIC. The preclinical evidence is encouraging. Multiple animal studies have demonstrated attenuation of doxorubicin-induced cardiomyocyte injury with both empagliflozin and dapagliflozin [18,19], and a large-animal swine model using a clinically translatable protocol showed that empagliflozin fully prevented AIC events while preserving myocardial energetics and mitochondrial structure [20]. These findings suggest an attenuation of cardiotoxicity in preclinical models.

Clinical data, however, are limited. No completed randomized controlled trials (RCTs) have been published in this specific setting, and the available evidence is entirely observational. This review synthesizes the available evidence and frames all findings as hypothesis-generating rather than practice-changing. 

## Review

Methods

A selective narrative literature review was conducted from inception through 28 February 2026, without language restriction. Data were extracted using a pre-piloted standardized form capturing the following domains (see Appendix for full details): study identity, country, data source, design; population characteristics (sample size, age, sex, cancer type, diabetes prevalence, anthracycline agent and dose, co-medications); SGLT2i agent, dose, and timing relative to chemotherapy; comparator; follow-up duration; outcome definitions and data source; outcome event counts or effect measures with variance. Databases searched included MEDLINE, Embase, Cochrane CENTRAL, ClinicalTrials.gov, Scopus, Web of Science Core Collection, and CINAHL using predefined keywords related to SGLT2i, anthracyclines, and cardiotoxicity.

Studies were eligible if they enrolled adults (≥18 years) with any histologically confirmed malignancy receiving anthracycline-based chemotherapy (doxorubicin, epirubicin, daunorubicin, idarubicin, or liposomal formulations) at any cumulative dose; examined any SGLT2i (empagliflozin, dapagliflozin, canagliflozin, ertugliflozin) at any dose during or after anthracycline treatment; compared against unexposed controls not receiving SGLT2i; and reported imaging-confirmed CTRCD, clinical HF hospitalization, all-cause mortality, composite MACE, or prespecified adverse events. RCTs, prospective cohort studies, retrospective cohort studies (including EHR/administrative database studies), and prospective non-randomized comparative studies were eligible. Case reports, letters without original data, narrative reviews, and secondary meta-analyses were excluded.

After handsearching and backward and forward citation tracking, we have identified six clinical studies, published between 2022 and 2024, which constitute the entire published evidence base on this question [21-26]. Together, they enrolled 21,523 patients in total across the United States, Canada, Taiwan, and Argentina, constituting a geographically diverse but methodologically homogeneous group. Five out of the six included studies were retrospective observational cohort studies. The sixth, EMPACARD-PILOT, was a prospective non-randomized comparative study from Argentina [26]. The findings were synthesized qualitatively to provide a comprehensive overview of current evidence.

All five retrospective cohorts enrolled patients with pre-existing type 2 diabetes exclusively. This reflects the clinical context at the time these datasets were assembled, when SGLT2i were primarily prescribed for glycemic control. However, two of the largest studies introduce an important limitation with respect to anthracycline specificity. Bhatti et al. [25] (n=17,350) and Avula et al. [24] (n=1,280) included patients receiving a broad range of cardiotoxic chemotherapy agents, with anthracyclines comprising only approximately 25% and 19% of exposures, respectively. As a result, the associations observed in these studies reflect a mixed cardiotoxic chemotherapy population rather than isolated anthracycline-induced cardiotoxicity. Their findings should therefore be interpreted as indirect evidence and not specific to anthracycline-related cardiac dysfunction, which limits alignment with the anthracycline-focused scope of this review.

Imaging-based evidence

Two studies assessed cardiac function using echocardiography-based endpoints, and both found directionally consistent results. Gongora et al. [21], using a composite imaging endpoint derived from electronic health record LVEF data at Massachusetts General Hospital, found that SGLT2i use was associated with substantially lower odds of CTRCD among 128 patients (OR 0.17; 95% CI 0.05-0.63). EMPACARD-PILOT [26] was more rigorous in its cardiac assessment relying on 2D echocardiography with GLS measurement in 76 breast cancer patients receiving doxorubicin at 60 mg/m² for four cycles and similarly found a lower incidence of CTRCD in the empagliflozin group (6.5% vs 35.5%; RR 0.18; 95% CI 0.04-0.75; p=0.005), with LVEF also significantly higher at six months in the treated group (56.8±5.8% vs 53.7±6.7%; p=0.029).

These estimates are not directly comparable due to differences in study design, populations, outcome ascertainment, and effect measures. What they share is a directional consistency: both point toward protection, and both report statistically significant associations, although based on small samples and/or non-randomized designs. Whether that consistency reflects a true biological signal or a shared susceptibility to confounding is a question that cannot be answered with these data.

EMPACARD-PILOT is the only prospective study in this evidence base. It uses a rigorous echocardiographic methodology. However, it also has significant limitations: the non-randomized design involved clinical phenotype-based group allocation; there was a significant imbalance in beta-blocker use at baseline (58% in the empagliflozin group vs 34% in controls, p=0.038), which is itself a recognized cardioprotective intervention in AIC; and with only 76 patients, the confidence intervals are wide [26]. These caveats do not negate the findings, but they prevent causal attribution.

Clinical event data

Four studies reported clinical endpoint data in forms suitable for time-to-event analysis. Gongora et al. [21] and Chiang et al. [22] reported hazard ratios for HF hospitalization and were the most clinically comparable pair. Both studies reported hazard ratios suggesting a reduction in HF hospitalization with SGLT2i use, although estimates were imprecise and varied across analyses.

When the larger administrative dataset by Bhatti et al. [25] was considered alongside these studies, the overall direction of association remained similar. However, interpretation is limited by the inclusion of mixed chemotherapy populations and the use of administrative outcome definitions.

Abdel-Qadir et al. [23] reported an unusual finding: zero HF hospitalization events in the 99 SGLT2i-treated patients over a median follow-up of 1.6 years, compared with 31 events in 834 controls. This is striking, but zero-event data from a small, exposed group is statistically weak. It reflects inadequate statistical power and a small sample size rather than a complete protective effect. A hazard ratio could not be estimated with conventional survival analysis methods.

Avula et al. [24] addressed a different question from the others: they enrolled patients with pre-existing CTRCD or HF at entry, rather than studying primary prevention. In this treatment context, SGLT2i use was associated with lower odds of HF exacerbation (OR 0.48; 95% CI 0.36-0.65). This finding aligns mechanistically with the established HF trials, but it represents a different clinical situation from the prevention-oriented studies.

Mortality

All-cause mortality data were reported by three studies, with hazard ratios generally favoring SGLT2i use. However, the magnitude of the observed associations is unlikely to be causal. Mortality is the outcome most susceptible to healthy user bias, channeling bias, and immortal time bias in pharmacoepidemiological research, and a nearly 50% mortality reduction over 12-18 months would be biologically extraordinary even if the cardioprotective mechanism were real. Thus, this signal is substantially or entirely driven by confounding.

The confounding problem

The observed effect sizes are unlikely to represent accurate estimates of the true treatment effect and are probably inflated.

In the HF trials, SGLT2i reduced HF hospitalizations by roughly 25-30% over 16-26 months in patients with established HF [12-15]. The reviewed observational studies report HRs of 0.25-0.51 for various clinical endpoints (protection of 49-75%). The gap between what is mechanistically plausible and what is statistically observed is a suggestive indicator that unmeasured confounding is contributing substantially to the apparent effect.

There are several specific bias mechanisms. Healthy user bias is probably the most pervasive: patients receiving SGLT2i are, by definition, under active metabolic management, typically by cardiologists or diabetologists who are also likely to prescribe other cardiovascular protective agents, ensure closer cardiac monitoring, and select patients with better overall functional status. None of these advantages can be fully captured in a propensity score model [23,24]. Immortal time bias arises when the period between the start of follow-up and the initiation of treatment, during which subjects cannot experience the outcome, is misclassified as treated time; patients who survive long enough to accumulate SGLT2i prescriptions will always appear to do better than those who die early. Protopathic bias may also operate in the opposite direction if physicians discontinue SGLT2i when early cardiac decompensation is detected, making treated patients appear healthier than they are at baseline.

None of these biases can be fully corrected post hoc in retrospective data, and they compound each other. The directional consistency of the signal across geographically and institutionally diverse study populations is hypothesis-generating. 

Mechanistic plausibility

Whatever the limitations of the observational data, the mechanistic case for SGLT2i in the anthracycline setting has become increasingly well-characterized and is biologically coherent.

The primary injury cascade in AIC involves topoisomerase IIβ inhibition leading to DNA double-strand breaks, p53 activation, and cardiomyocyte apoptosis, compounded by oxidative stress from anthracycline-iron quinone redox cycling [5,6]. SGLT2i engage several complementary protective mechanisms that address different points in this cascade. AMPK activation, a consistent cellular response to SGLT2i, promotes mitochondrial biogenesis and autophagic clearance of damaged organelles, both of which are impaired in anthracycline-exposed cardiomyocytes [16]. NLRP3 inflammasome suppression has been demonstrated in preclinical AIC models [18], addressing the sterile inflammatory component of myocardial injury. NHE1 inhibition prevents the sodium and calcium overload that develops following cardiomyocyte energetic compromise [17], and the metabolic shift toward ketone body utilization provides an alternative energy source for the myocardium under bioenergetic stress [27].

The large-animal data from Medina-Hernández et al. [20], using a clinically translatable swine protocol, are particularly compelling: empagliflozin fully prevented AIC events in a dose-dependent fashion while preserving mitochondrial structure. These data provide biologically plausible support for a potential cardioprotective effect.

This mechanistic coherence does not prove clinical efficacy; many mechanistically plausible interventions have failed in randomized trials, but it provides a legitimate biological framework for future studies [8-11].

The heterogeneity of outcomes

One of the methodological challenges is the inconsistency in how cardiac dysfunction is defined and measured. Five of the six studies relied on administrative ICD coding for outcome ascertainment, without echocardiographic data. Administrative definitions have known limitations: they tend to capture overt, symptomatic events while missing the earlier, subclinical dysfunction that contemporary cardio-oncology guidelines, including the 2022 European Society of Cardiology (ESC) Cardio-Oncology Guidelines [28], emphasize as the primary target of surveillance and intervention. The imaging-based threshold of a ≥10% decline in LVEF to below 50%, or a ≥15% relative decline in GLS, is sensitive to subclinical injury in a way that a hospitalization code simply is not.

This means the administrative studies and the imaging studies are measuring different things. The former captures more severe, end-stage events and the latter detects earlier dysfunction that may be more reversible. The fact that directional consistency is maintained across both types of endpoints could be interpreted as strengthening the biological plausibility of the signal: if SGLT2i were simply preventing overt HF hospitalizations through some administrative coding artifact, one would not expect to see the same pattern in dedicated echocardiographic studies. But it also means direct comparisons between studies should be made carefully.

Notably, none of the retrospective studies measured GLS, which is recognized as a more sensitive early marker of subclinical dysfunction than LVEF [29,30]. Only EMPACARD-PILOT incorporated GLS measurement [26]. Any future prospective study in this area should use GLS, not only LVEF, as the primary imaging endpoint [29,30].

This heterogeneity limits quantitative comparison across studies and further reinforces the need for standardized, imaging-based endpoints in prospective trials.

Safety considerations

Across all six studies, SGLT2i use in cancer patients was not associated with a clinically significant increase in serious adverse events. Rates of euglycemic diabetic ketoacidosis, urinary tract infections, genital mycotic infections, and hypotension were not observed to be elevated, although adverse event ascertainment in observational datasets may be incomplete. These findings are consistent with broader cardio-oncology safety analyses suggesting that SGLT2i are generally well tolerated in cancer populations, with manageable risks when appropriate patient selection and monitoring strategies are applied [31].

Institutional protocols for SGLT2i interruption, typically 3-4 days before planned procedures, will need to be adapted to the oncological context, where surgical and procedural timelines may be less predictable [31]. But the consistent safety signal across nearly 21,523 patients supports the clinical feasibility of SGLT2i use in this population and should not be a barrier to the ongoing RCTs.

The ongoing randomized trials

Three adequately designed prospective RCTs are now actively recruiting, and they represent the appropriate pathway to resolve the question that observational data cannot answer. EMPACT (NCT05271162) is assessing empagliflozin 10 mg/day versus placebo in non-diabetic patients receiving anthracyclines, with LVEF and GLS as primary endpoints over 12 months; notably, it addresses the non-diabetic population. PROTECT (NCT06341842) is evaluating dapagliflozin versus placebo in early-stage breast cancer patients receiving anthracyclines and/or trastuzumab, with an 18-month follow-up. PROTECTAA (NCT06304857) is focused specifically on dapagliflozin in breast cancer patients receiving anthracyclines [32,33].

These trials share some important design features that distinguish them from the observational literature: they are not restricted to diabetic patients; they use imaging-based primary endpoints; and they are prospectively controlled. The results will determine whether the observational signal reflects a cardioprotective effect or a statistical mirage.

## Conclusions

The intersection of SGLT2i pharmacology and anthracycline cardiotoxicity represents a biologically plausible therapeutic hypothesis supported by consistent observational signals across six studies including 21,523 patients. However, a key insight from this review is that, despite directional consistency across heterogeneous populations and study designs, the magnitude of the observed associations appears disproportionately large compared with established HF trials. This discrepancy suggests that residual confounding, including healthy user and immortal time bias, is likely contributing substantially to the apparent benefit. In addition, the evidence base is largely restricted to patients with type 2 diabetes and frequently relies on administrative outcome definitions, limiting generalizability and mechanistic interpretation.

Definitive clinical evidence remains forthcoming. Ongoing randomized trials, including EMPACT, PROTECT, and PROTECTAA, will determine whether these observational signals translate into true cardioprotective effects. Until such data are available, routine use of SGLT2i for CTRCD prevention cannot be recommended, and their use should be restricted to clinical trial settings.

## Appendices

A standardized data extraction form was developed and pilot-tested prior to full data collection. The form was designed to ensure consistency and completeness across studies and included the following variables:

Study characteristics

-First author, year of publication

-Country

-Study design (retrospective cohort, prospective cohort, non-randomized study)

-Data source (e.g., administrative database, electronic health record, single-center cohort)

Population characteristics

-Sample size

-Mean or median age

-Sex distribution

-Cancer type

-Prevalence of type 2 diabetes mellitus

-Anthracycline agent(s) and cumulative dose (when available)

-Concomitant cardiovascular medications (e.g., beta-blockers, RAAS inhibitors)

Exposure details

-SGLT2 inhibitor agent

-Dose (when reported)

-Timing of initiation relative to chemotherapy

Comparator

-Description of control group (non-exposed or alternative therapy)

Outcomes

-Definition of cancer therapy-related cardiac dysfunction (CTRCD)

-Imaging-based outcomes (LVEF, GLS)

-Clinical outcomes (heart failure hospitalization, mortality, composite endpoints)

-Data source for outcome ascertainment

Follow-up and effect measures

-Duration of follow-up

-Effect estimates (hazard ratios, odds ratios, relative risks)

-Measures of variance (confidence intervals, standard errors)

The form was iteratively refined during pilot testing to ensure clarity and applicability across different study designs.
